# Ultrasound-guided microwave ablation for the treatment of idiopathic granulomatous mastitis: comparison with surgical excision

**DOI:** 10.1186/s12905-024-03070-7

**Published:** 2024-04-18

**Authors:** Hang Li, Guoliang Zhang, Hongling Wang, Haiying Chen, Xiaoli Liu, Chuansheng Zheng, Lisheng Lin, Lihong Li

**Affiliations:** 1 The School of Clinical Medicine, Fujian Medical University, Fujian, 350000 China; 2 Department of Breast Surgery, Affiliated Hospital of Putian University, Fujian, 351100 China; 3 Department of Thyroid Surgery, Affiliated Hospital of Putian University, Fujian, 351100 China; 4 Department of General Surgery, Xiamen Xinkaiyuan Hospital, Fujian, 361000 China; 5 Department of Pathology, Affiliated Hospital of Putian University, Fujian, 351100 China

**Keywords:** Idiopathic granulomatous mastitis (IGM), Microwave ablation, Ultrasound, Thermal therapy

## Abstract

**Background:**

Idiopathic granulomatous mastitis (IGM) results in notable clinical symptoms and breast deformity. This study aimed to evaluate the clinical feasibility of microwave ablation (MWA) for the treatment of IGM through comparison with surgical excision.

**Methods:**

From June 2016 to December 2020, a total of 234 consecutive patients admitted to the hospital were retrospectively included in this study. IGM was pathologically confirmed via breast biopsy in all included patients. These patients were divided into the MWA group (*n* = 91) and surgical group (*n* = 143) based on the type of treatment. Patients in both groups received oral prednisone prior to intervention. The clinical remission rate, recurrence rate, operative pain, complications, and BREAST Q score were compared between the two groups.

**Results:**

There were 340 lesions in the MWA group, and 201 lesions in the surgical group were ultimately included. Significant differences in the complete remission rate (96.7% vs. 86.7%, *p* = 0.020), recurrence rate (3.3% vs. 13.3%, *p* = 0.020), operation time (48.7±14.6 min vs. 68.1±36.4 min, *p* < 0.001), postoperative pain (*p* < 0.001) and postoperative BREAST Q score (*p* < 0.001) were observed between the MWA and surgical groups.

**Conclusions:**

Microwave ablation is feasible for the treatment of IGM, due to its high curative rate and low recurrence rate. Because of the minimal invasiveness of MWA and sufficient preservation of the gland and contour of the breast, patients are more satisfied with the appearance of the breast. Therefore, for patients with complex conditions requiring surgery, MWA is a good alternative treatment.

## Introduction

Idiopathic granulomatous mastitis (IGM) is a class of chronic inflammatory mammary disease that involves granuloma formation and noncaseous necrosis in the lobules of the breast [1]. IGM is prevalent among young and middle-aged women with a history of childbearing and lactation [2]. Multiple aetiologic factors, including infection, hyperprolactinemia, oral contraceptive pill use, milk deposition and autoimmune response, have been reported to be responsible for IGM [3–7]. This disease manifests a variety of symptoms, such as a breast mass, abscess, pain, rubefaction and fistula [1, 8, 9]. Because of the notable clinical symptoms and breast deformities, an effective treatment method is required for IGM patients.

Treatments, including antibiotics, surgical interventions, and immunosuppressants, have been applied for treating of IGM [10, 11]. However, no consensus has been reached about the optimal therapeutic methods. Although studies have reported that some IGM cases tend to resolve spontaneously without any intervention [12, 13], a long recovery process may lead to changes in breast appearance and texture [1], and patients who do not undergo remission may develop disease progression, such as enlarged lesions, abscesses, skin ulcers, and sinus tract formation. Treatment with steroids or methotrexate may generate drug resistance and potential side effects [14, 15]. Surgical interventions, including abscess incision, drainage, and extended resection, have been applied for IGM with different manifestations, whereas recurrence events occur in some cases [8, 16, 17], and extended mammary lesion excision is destructive to the function and cosmetic appearance of the breast [13].

Microwave ablation (MWA) is regarded as a minimally invasive method that is widely used for the treatment of various benign and malignant lesions in a variety of organs [18–23]. Microwaves generate power through vibrating water molecules and heat the surrounding tissue of the electrode to generate high focal temperatures, leading to protein coagulation and degradation, through which MWA causes lesion tissue coagulative necrosis and destroys nodular lesions via the thermal energy of microwaves [23, 24]. However, studies on the application of MWA for IGM treatment are rare [1]. More clinical evidence is needed to demonstrate the importance of MWA for the treatment of IGM. This study aimed to explore the feasibility of MWA for the treatment of IGM by comparing MWA with surgical excision.

## Patients and methods

### Research population

**Fig. 1:**
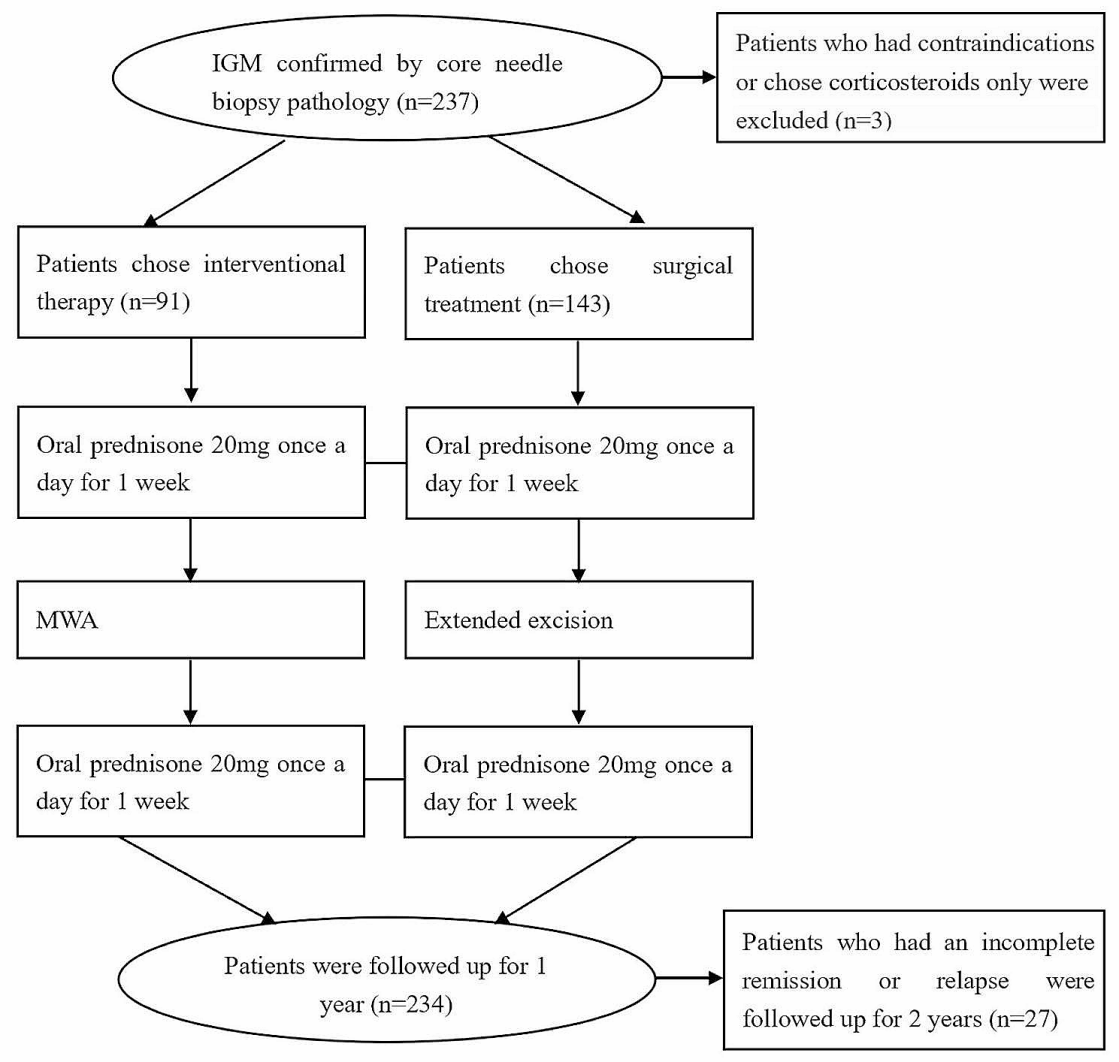
Patient enrolment and management process

**Fig. 2:**
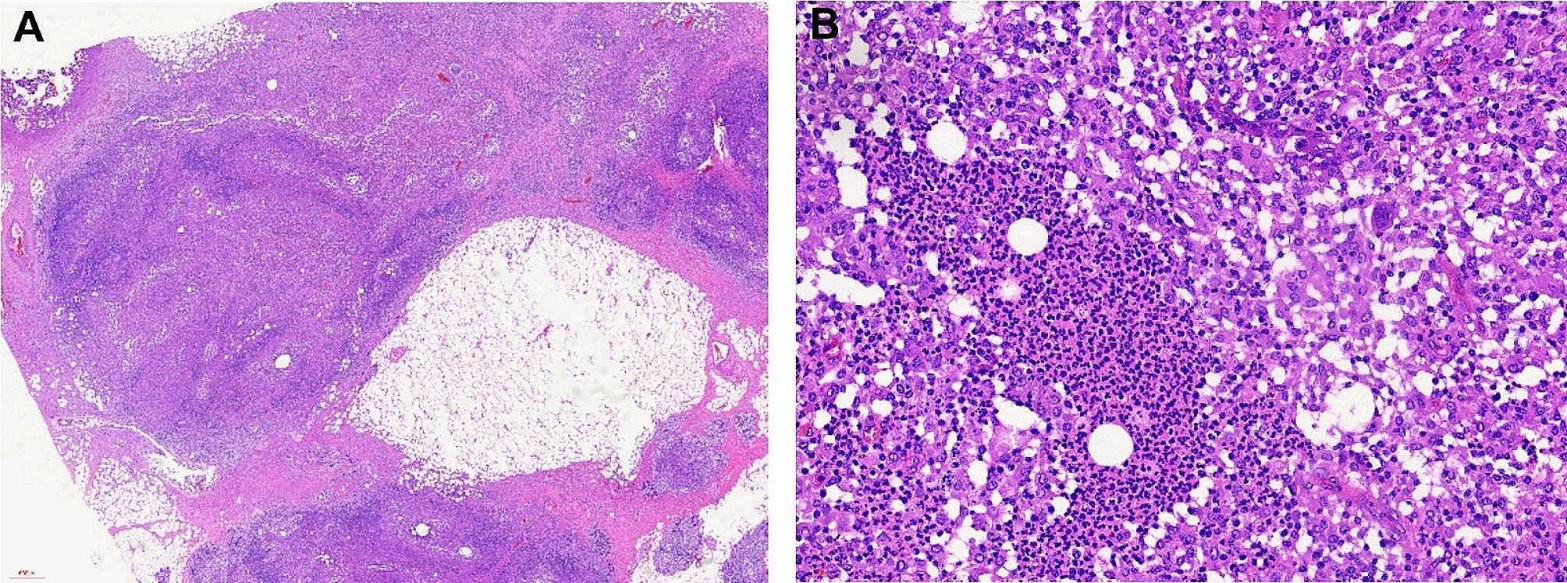
Pathological diagnosis of IGM by core needle biopsy. (**A**) Magnification 20×. (**B**) Magnification 400×

Among the patients who had been diagnosed with IGM using ultrasound-guided breast biopsy from June 2016 to December 2020 in our hospital, 234 patients were retrospectively included in this study. Therapies of all the patients were made according to their individual condition and preferences. Ultimately, 91 patients treated with ultrasound-guided microwave ablation plus prednisone were included in the MWA group, and 143 patients treated with extended excision plus prednisone were included in the control group (surgical group). The inclusion of patients and the management process are shown in Fig. 1. The pathological diagnosis of IGM is shown in Fig. 2.

Patients who met the following criteria were enrolled: (1) were aged older than 18 years; (2) had clinical manifestations, physical examinations or ultrasonography findings predicting the presence of lesions in the breast; and (3) had a breast pathological diagnosis of IGM by ultrasound-guided core needle biopsy, with the typical pathology of noncaseating necrotic granulomas or microabscesses present in the lobules of the breast [25].

Patients with any of the following conditions were excluded: (1) severe coagulation disorders; (2) acute infectious diseases; (3) conditions that prohibited the use of glucocorticoids, such as diabetes, liver and kidney insufficiency, and viral hepatitis; (4) breast cancer; or (5) pregnancy or lactation.

This study was approved by the Ethics Committee of our hospital. All patients provided informed consent for our use of their clinical data.

### Research methods

After confirmation of IGM, patients were prescribed oral prednisone acetate tablets (20 mg once a day) for one week and then admitted to the hospital. For patients with mammary abscess, pus was aspirated for aetiological examination before intervention. Antibiotics were only used for patients who tested positive for bacterial cultures.

#### MWA group

**Fig. 3:**
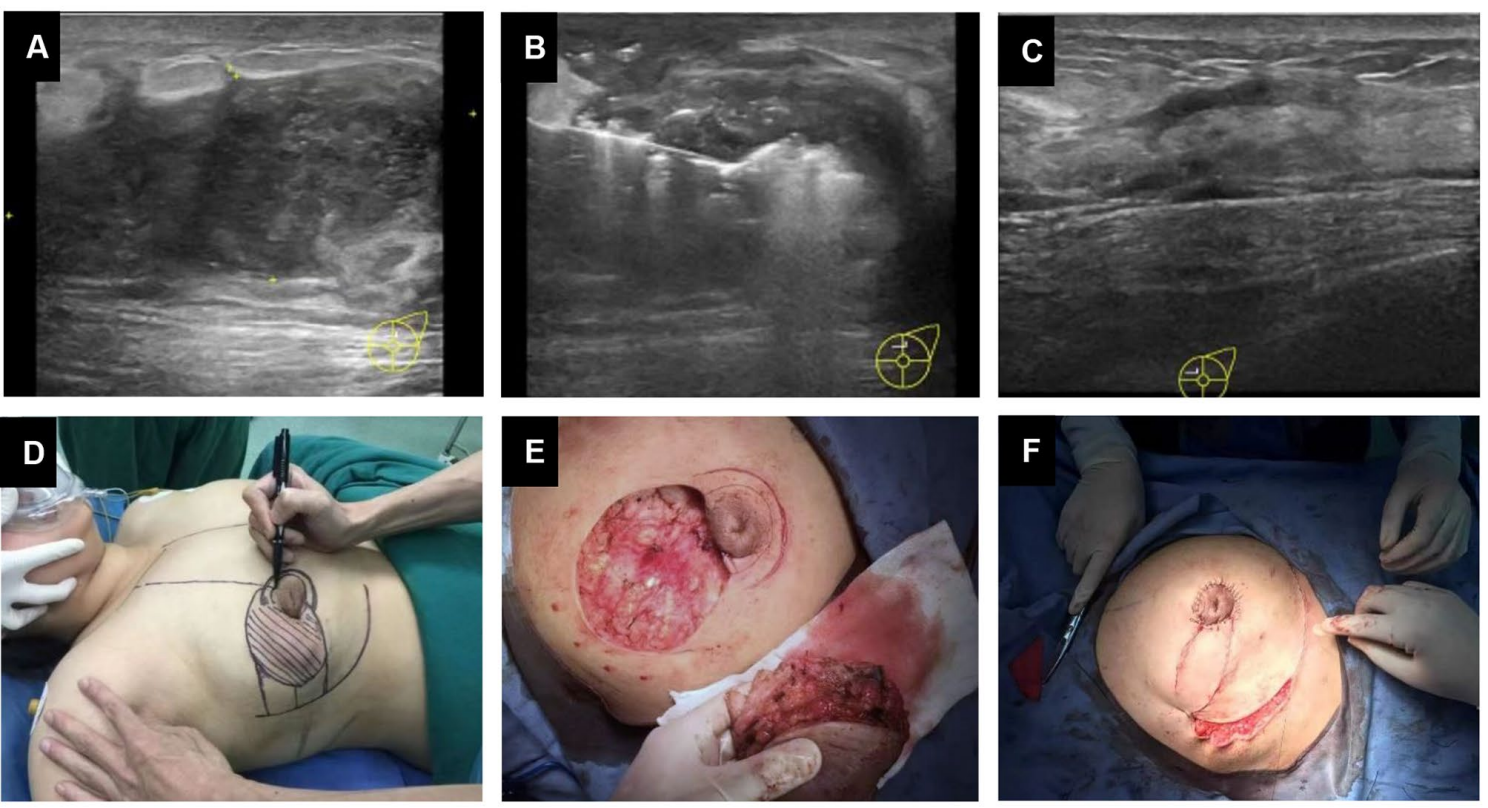
The operative processes of the two groups. (**A**) Preoperative ultrasound localization. (**B**) Ultrasound-guided focal ablation. (**C**) Postoperative ultrasound examination. (**D**) Determination of surgical methods. (**E**) Extended resection of lesions. (**F**) Breast reconstruction

Ultrasound was used to determine the characteristics of the lesions, such as their location, size, shape, amount, echogenicity and blood flow signals. An individualized ablation plan, including the puncture site, depth of needling, necessity of abscess aspiration, and ablation duration, was made based on the preoperative ultrasound results. The patients were placed in a supine position with their arms outstretched and their hands placed behind the occiput. Local infiltration anaesthesia was applied under the skin and the retromammary space of the breast. For lesions adjacent to the skin, areola, and/or pectorales less than 1 cm, 0.9% saline was injected subcutaneously and/or into the retromammary space to create a barrier to prevent thermal damage. For the centre area of the lesions, an output power of 35 W was set for ablation, while for the periphery of the lesions, an output power of 25 W was set for ablation. Fixed-point ablation was used for microabscesses, and mobile ablation was required for multiple lesions and large lesions with a maximum diameter > 5 cm. The ablation process was performed from deep to shallow and distal to proximal. Complete ablation was indicated when the ultrasound signal of the whole lesion became hyperechoic. Then, patients were treated with local dressings and ice packs and discharged 8 h after intervention. Oral prednisone acetate tablet treatment was continued for one week postoperatively. The ultrasound results before and after ablation are shown in Fig. 3A and C.

#### Surgical group

After routine preoperative preparation, the patients were placed in a supine position with their arms outstretched. Then, general anaesthesia was applied. Extended resection was applied to remove the lesion thoroughly, including the affected skin, inflammatory or necrotic tissues, breast abscess and sinus track. For patients with breast defects larger than 5 cm in diameter or more than 30% of the gland removed, breast reconstruction with a random breast dermoglandular flap (BDGF) was performed (Fig. 3D and F). Patients were discharged from the hospital after examination without infection or seroma at 48 h after surgery. Oral prednisone acetate tablet treatment was continued for one week postoperatively.

### Clinical parameters

Clinical data, including the clinical remission rate, complete remission rate, recurrence rate, and complete remission time, were recorded to assess therapeutic efficacy. The operation time was recorded to assess the complexity of the procedure. Postoperative pain was recorded to assess operative injury. The incidence of complications was recorded to assess the safety of the treatments. For patients in both the MWA group and surgical group, physical examination and breast ultrasonography were used to assess clinical remission. Postoperative pain was graded as follows: degree 0 (no pain), degree I (mild pain, without pain medication), degree II (moderate pain, impact rest, requires pain medication), and degree III (severe pain, persistent pain, requires stronger analgesics for pain relief). Patients who met the following criteria were considered to achieve complete remission: (1) had no lesion detected on ultrasound and a pathological diagnosis of the ablated site without typical pathological features; and (2) did not require additional MWA treatment, oral prednisone therapy, or surgical excision. Patients who met the following criteria were considered to achieve clinical remission: (1) clinical symptom remission, including no pain, skin ulcer or sinus healing, and skin redness subsiding; and (2) shrinkage of lesions in the breast on the ultrasound. The BREAST Q questionnaire (copyright of Memorial Sloan Kettering Cancer Center and The University of British Columbia) was administered preoperatively and at 6 months postoperatively to survey patient satisfaction with the breasts.

### Follow-up plan

All patients were followed up according to a uniform follow-up plan. In the first 6 months after treatment, patients were followed up monthly, and physical examination or ultrasound examination was performed. At 6 to 12 months after treatment, patients were followed up every three months, and physical examination and ultrasound examination were performed to track the patient’s recovery. For patients whose disease failed to complete remission, follow-up was extended to 24 months. At 6 and 12 months after treatment, core needle biopsy was used to evaluate pathological alterations in patients with incomplete resolution of the lesion in the MWA group or with new lesions detected in both groups.

### Statistics

SPSS Statistics for Windows, version 22.0 (SPSS Inc., Chicago, IL, USA) was used to process the data for statistical analysis. Measurement data are presented as the mean (standard deviation), and Student’s t test was applied to evaluate the differences between the groups. Count data were tested by Pearson’s χ2 test or Fisher’s exact test. *P* < 0.05 was considered to indicate statistical significance.

## Results

There were 340 lesions ablated in the MWA group, and 201 lesions were ultimately resected in the surgical group. There was a significant difference in lesion number and operation time between the two groups (*p* < 0.001). No significant differences were observed in sex, age, lesion size, lesion distribution, skin involvement ratio, abscess formation ratio, or sinus formation ratio between the two groups (Table 1). All the treatments and follow-ups were implemented successfully according to our program.

**Table 1:** Characteristics of the enrolled patients (*n* = 234)

Parameters	MWA group(*n* = 91)	Surgical group(*n* = 143)	t/χ^2^	*p*-value
Age, year [mean, (SD)]	32.4(6.6)	34.2(8.6)	1.83	0.069
Maximum diameter, mm [mean, (SD)]	36.8(15.1)	33.6(23.6)	1.24	0.218
Lesion number, n (%)	3.7(4.0)	1.4(0.7)	5.54	< 0.001
Lesion distribution, n (%)			3.83	0.148
Left side	37(40.7)	68(47.6)		
Right side	51(56.0)	64(44.8)		
Both sides	3(3.3)	11(7.7)		
Lesion location, n (%)			0.44	0.505
Single quadrant	61(67.0)	103(72.0)		
Multiple quadrants	30(33.0)	40(28.0)		
Skin involvement, n (%)			0.01	0.913
Yes	32(35.2)	48(33.6)		
No	59(64.8)	95(66.4)		
Abscess formation, n (%)			1.68	0.195
Yes	41(45.1)	51(35.7)		
No	50(54.9)	92(64.3)		
Sinus track formation, n (%)			< 0.01	1.000
Yes	1(1.1)	1(0.7)		
No	90(98.9)	142(99.3)		
Operation time (mean, SD)	48.7(14.6)	68.1(36.4)	5.68	< 0.001

SD: Standard deviation. *P* < 0.05 was considered to indicate statistical significance

### Efficacy assessment

We compared the clinical remission rate, complete remission rate, and recurrence rate to assess the efficacy of the treatments in the two groups. Since rehabilitation after microwave ablation is an ongoing process, complete remission time was recorded only for the MWA group. Clinical manifestations such as pain, abscess, rubefaction, skin ulcer and/or sinus track were relieved significantly in 89 patients in the MWA group, resulting in a clinical remission rate of 97.8%. In the surgery group, 140 patients experienced relief immediately after completing postoperative oral drug therapy, resulting in a clinical remission rate of 97.9%. Ultrasonography revealed that the lesions decreased gradually and were completely absorbed in 88 patients in the MWA group after an average duration of 10.8±2.7 months, with a complete remission rate of 96.7%, while in the surgical group, 124 patients achieved complete remission, with a rate of 86.7%. However, 3 patients in the MWA group and 19 in the surgical group relapsed during subsequent follow-up, with recurrence rates of 3.3% and 13.3%, respectively. There was a significant difference in the complete remission rate (*p* = 0.020) and recurrence rate (*p* = 0.020) between the two groups (Table 2). Furthermore, we conducted a stratified chi-square test to explore the risk factors correlated with complete remission in the two groups. The results showed that patients in the MWA group with IGM distributed in multiple quadrants had better complete remission than did those in the surgical group (*p* = 0.012, Table 3).

**Table 2:** Comparisons of therapeutic efficacy between the two groups

	MWA group(*n* = 91)	Surgical group(*n* = 143)	*p*-value
Clinical remission, n (%)	89(97.8)	140(97.9)	1.000
Complete remission, n (%)	88(96.7)	124(86.7)	0.020
Recurrence, n (%)	3(3.3)	19(13.3)	0.020
Complete remission time, months [mean, (SD)]	10.8(2.7)	/	/

MWA: microwave ablation. SD: Standard deviation. *P* < 0.05 was considered to indicate statistical significance

**Table 3:** Stratified analysis of risk factors influencing complete remission in the two groups

	Complete remission, n (total)		*p*-value
	MWA group, *n* = 88(91)	Surgical group, *n* = 124(143)	
Skin involvement			
Yes	30(32)	34(44)	0.104
No	58(59)	90(99)	0.092
Abscess formation			
Yes	40(42)	42(51)	0.104
No	48(49)	82(92)	0.097
Lesion location			
Single quadrant	60(61)	98(103)	0.413
Multiple quadrants	28(30)	26(40)	0.012
IGM stage			
Mass stage	48(49)	82(92)	0.096
Abscess formation stage	38(39)	39(46)	0.065
Refractory stage	2(3)	3(5)	1.000

MWA: microwave ablation. *P* < 0.05 was considered to indicate statistical significance

### Postoperative pain

In the MWA group, 24 patients had no pain, 57 patients had mild pain, 10 patients had moderate pain, and no patient had severe pain in the ablation area. In the surgical group, 18 patients had no pain, 76 patients had mild pain, 43 patients had moderate pain, and 6 patients had severe pain in the surgical area. There were significantly more patients with no pain and fewer patients with moderate pain in the MWA group than in the surgical group (*p* < 0.05). The results indicated that MWA caused less operative trauma in treating IGM (Table 4).

**Table 4:** Comparisons of postoperative pain

	MWA group(*n* = 91)	Surgical group(*n* = 143)	*p*-value
Pain			< 0.001
No, n (%)	24(26.4)	18(12.6)	0.012
Mild, n (%)	57(62.6)	76(53.1)	0.196
Moderate, n (%)	10(11.0)	43(30.1)	0.001
Severe, n (%)	0(0)	6(4.2)	0.084

MWA: microwave ablation. *P* < 0.05 was considered to indicate statistical significance

### Assessment of complications

In the MWA group, 1 patient had incision/ablation site fat liquefaction, 2 patients had skin heat injury, 1 patient developed a sinus tract at the puncture site, and 1 patient developed skin ulceration. Five patients in the MWA group developed complications, resulting in an incidence rate of 5.5%. In the surgical group, 1 patient had an incision haematoma, which was properly resolved by local compression, haemostatic medication, and aspiration. In addition, 10 patients developed breast deformation, 5 patients developed nipple deformation, 4 patients developed scar hyperplasia, and 3 patients developed lactation disorders. In total, there were 23 patients in the surgical group who developed complications, resulting in an incidence rate of 16.1%. The MWA group had a significantly lower rate of overall complications than the surgical group (Table 5).

**Table 5:** Complications in the two groups

	MWA group(*n* = 91)	Surgical group(*n* = 143)	*p*-value
Complications, n (%)			
Sinus tract formation	1(1.1)	0(0)	
Fat liquefaction	1(1.1)	0(0)	
Skin heat injury	2(2.2)	0(0)	
Skin ulceration	1(1.1)	0(0)	
Incision haematoma	0(0)	1(0.7)	
Breast deformation	0(0)	10(7.0)	
Nipple deformation	0(0)	5(3.5)	
Scar hyperplasia	0(0)	4(2.8)	
Lactation disorder	0(0)	3(2.1)	
Total	5(5.5)	23(16.1)	0.026

MWA: microwave ablation. *P* < 0.05 was considered to indicate statistical significance

### Cosmetic appearance and breast satisfaction

**Fig. 4:**
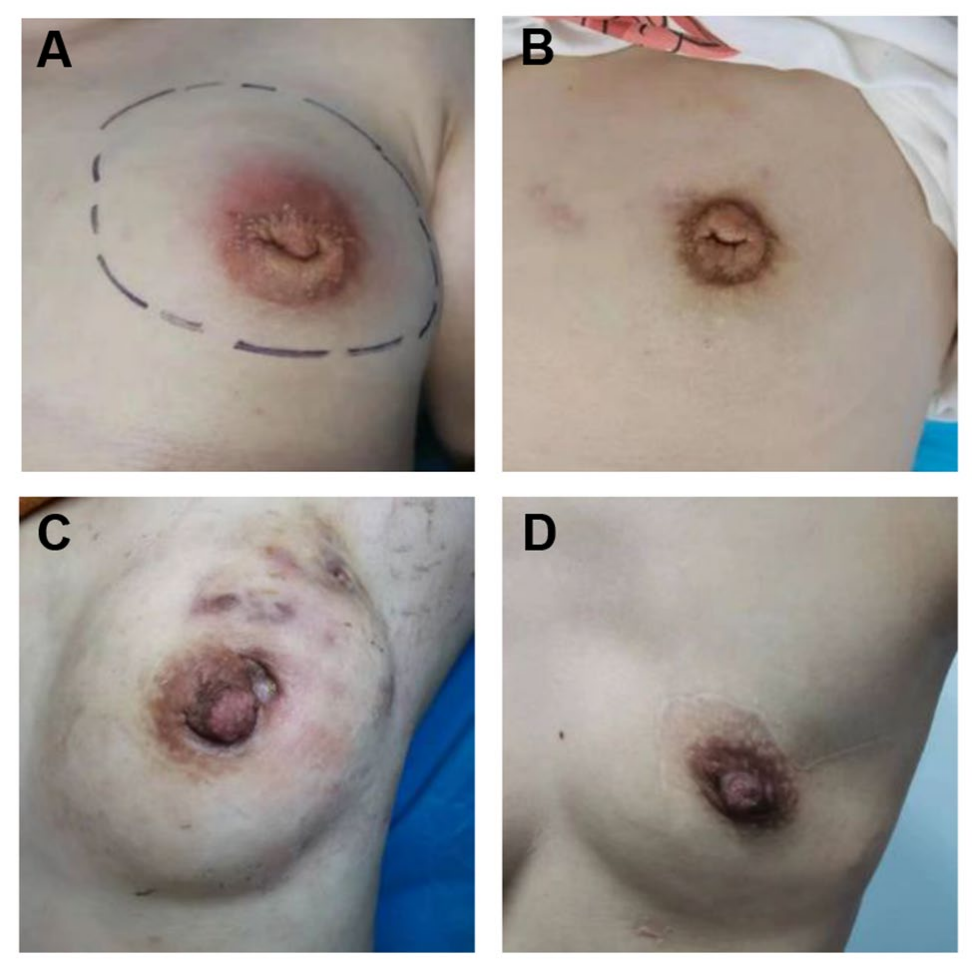
Comparisons of breast appearance between the two groups after treatment. (**A**) Breast appearance before MWA. (**B**) Breast appearance after MWA. (**C**) Breast appearance before surgery. (**D**) Breast appearance after surgery

We surveyed cosmetic appearance and breast satisfaction by the BREAST Q score questionnaire preoperatively and at 6 months postoperatively. There were no statistically significant differences between the two groups in terms of preoperative scores of satisfaction with breasts, psychosocial well-being, or physical well-being. However, after the operation, the scores of satisfaction with the breasts were 78.7±6.9 in the MWA group and 63.9±7.7 in the surgical group, the psychosocial well-being scores were 92.3±2.9 in the MWA group and 83.5±8.3 in the surgical group, and the physical well-being scores were 92.9±5.3 in the MWA group and 84.1±4.0 in the surgical group. There were significant differences in the scores for satisfaction with breasts (*p* < 0.001), psychosocial well-being (*p* < 0.001), and physical well-being (*p* < 0.001) between the two groups (Table 6). Comparisons of breast appearance before and after treatment are shown in Fig. 4A and D. Our analysis demonstrated that patients in the MWA group had a better cosmetic appearance than did those in the surgical group.

**Table 6:** Comparisons of the BREAST-Q scores between the two groups

	MWA group(*n* = 91)	Surgical group(*n* = 143)	*p*-value
**Preoperative**			
Satisfaction with Breasts	78.2±7.5	76.8±8.6	0.216
Psychosocial Well-being	92.6±3.5	92.4±3.9	0.682
Physical Well-being	87.0±6.5	86.7±5.4	0.197
**Postoperative**			
Satisfaction with Breasts	78.7±6.9	63.9±7.7	< 0.001
Psychosocial Well-being	92.3±2.9	83.5±8.3	< 0.001
Physical Well-being	92.9±5.3	84.1±4.0	< 0.001

MWA: microwave ablation. *P* < 0.05 was considered to indicate statistical significance

## Discussion

Currently, the treatment of IGM is controversial [26]. In this study, we found that the MWA group had better disease remission, better breast appearance, a lower recurrence rate and less trauma than did the surgical group. Ultrasound-guided MWA is more beneficial than extended excision for the treatment of IGM. These findings may provide insight into alternative therapeutic methods for treating IGM.

In this study, we compared the clinical remission rate and curative efficacy between the MWA group and surgical group. Breast ultrasound and postoperative pathological examinations were performed to assess the curative efficacy of the treatments. We found that clinical remission in the MWA group was similar to that in the surgical group, but complete remission in the MWA group was significantly better than that in the surgical group, and the recurrence rate in the MWA group was significantly lower than that in the surgical group, indicating that the efficacy of MWA was better than that of extended resection. The stratified chi-square test suggested that MWA was preferable to surgical excision for the treatment of IGM distributed in multiple quadrants. The probable reason is that intraoperative ultrasound supervision contributes to detecting more microscopic lesions, making microwave ablation more thorough, whereas surgical resection tends to overlook some distant microscopic lesions because there is no real-time positioning by ultrasound equipment. A previous study by our department showed that ultrasound-guided microwave ablation, which is closely related to the precise positioning and good operational flexibility of ultrasonic monitoring, is beneficial for the treatment of multiple benign breast nodules [23]. Under the guidance of ultrasonic equipment, the microwave ablation electrode can accurately reach the lesion and fully ablate the lesions by adjusting the electrode and performing multipoint ablation. Even for multiple breast lesions, microwave ablation can also effectively eliminate and minimize breast tissue damage. Therefore, ultrasound-guided MWA is a precise and definitive treatment.

Previous studies have reported that microwave ablation has a shorter ablation time and larger ablation range than other ablation methods [27, 28]. In the comparison of the operative time between MWA and surgical resection, we found that MWA had a shorter operative time than surgical resection, which means that microwave ablation is simpler and has a shorter duration of surgical injury. After comparing the postoperative pain between the two groups, we found that the ratio of no pain to mild pain was higher in the microwave ablation group and that the ratio of moderate to severe pain was higher in the surgery group. The difference in operation time and operative pain between the two groups was significant. A short operative time is also correlated with little trauma [29]. In terms of pain and operation time, MWA is less traumatic than surgical resection for the treatment of IGM.

Surgical excision usually includes both the intact lesion and the affected skin. For larger and multiple lesions, large chunks of breast tissue are usually removed [8, 26], which can cause complications, including incision haematoma, deescalation, breast deformation, scar hyperplasia, and nipple deformation. Our study revealed a total incidence of complications of 16.1% in the surgical group. In the MWA group, complications, including sinus tract formation, skin heat injury, fat liquefaction and skin ulceration, occurred, with a total incidence rate of 5.5%. The difference in the incidence of complications between the two groups was significant. In addition, the anaesthesia used in the two treatments is also a major difference. MWA was performed under local anaesthesia, whereas extended excision was performed under general anaesthesia. Compared with general anaesthesia, local anaesthesia is obviously safer and less risky. The results suggest that MWA has fewer and milder complications and is safer than surgical resection. The complications of microwave ablation are mild and manageable. We utilized local disinfection and cold compresses to treat skin burns and achieved excellent recovery by preventing infection and scar formation. For the treatment of sinus tracts, wound dressing changes and drainage of the sinus tract were utilized, and good healing was achieved within one month. For the treatment of fat liquefaction, disinfection and dressing treatment were adopted. All the complications caused by MWA were well managed.

The BREAST-Q is a scale used to evaluate the health-related quality of life and satisfaction of patients undergoing breast surgery [30]. The scope of application includes breast augmentation, breast reduction, breast reconstruction, breast preservation and mastectomy [30]. We used this scoring system to evaluate patient satisfaction with the breast after surgery. Our analysis suggested that MWA has a notable advantage in maintaining breast appearance and function owing to its minimal invasiveness. Although complete excision can be used to thoroughly remove the lesion and relieve the disease effectively [8], patient satisfaction with the treatment decreases due to shape alterations attributed to cavity collapse of the breast and visible surgical scarring. Lin et al. [1] reported that MWA is effective in reducing the size of IGM lesions and preserving normal tissue, thereby greatly protecting the appearance of the breast. Hence, minimally invasive MWA is an appropriate option for the treatment of IGM.

The main limitation of the study is that the main treatments for IGM are medical, surgery is applied for patients with large lesions or abscesses, skin redness, ulcers, and fistula formation. A comparison between MWA or surgery and steroid therapy should be performed to evaluate the efficacy of IGM. However, in our department, steroid therapy is mainly used in mild patients, while MWA and surgery are used in patients with large lesions and complications, which makes it impossible for us to perform this comparison. Moreover, this was a retrospective study with limited available data, and comparisons between the two treatments were not sufficient. Some observation indicators, such as postoperative complications, were not comparable between the two groups, which made it difficult to accurately evaluate the difference between the two treatments in terms of complications.

## Conclusions

In summary, ultrasound-guided MWA is a feasible method for the treatment of IGM, especially complex IGM, with many advantages, such as better appearance, less invasiveness, and fewer complications, compared to those of surgical treatment. Thus, MWA is a good alternative treatment for patients requiring surgical intervention.

## Data Availability

The analysed datasets generated during the study are available from the corresponding author upon reasonable request.

## Abbreviations

BDGF: Breast dermoglandular flap
IGM: Idiopathic granulomatous mastitis
MWA: Microwave ablation
